# Operative versus nonoperative management of acute Achilles tendon rupture: a systematic review and meta-analysis of clinical outcomes from randomized controlled trials

**DOI:** 10.1080/07853890.2025.2537349

**Published:** 2025-11-17

**Authors:** Shenghao Xu, Jianlin Xiao, Yang Li, Enbo Liu, Yanguo Qin, Xianyue Shen

**Affiliations:** ^a^ Department of Orthopedics, China-Japan Union Hospital of Jilin University, Changchun, Jilin, China; ^b^ Department of Orthopedics, The Second Hospital of Jilin University, Changchun, Jilin, China; ^c^ Department of Orthopedics, Centre for Leading Medicine and Advanced Technologies of IHM, The First Affiliated Hospital of USTC, Division of Life Sciences and Medicine, University of Science and Technology of China, Hefei, Anhui, China

**Keywords:** Achilles tendon rupture, Operative, Nonoperative, Re-rupture, Complication, Meta-analysis

## Abstract

**Objective:**

The objective of this study was to compare the differences in re-rupture rates, complications, and functional assessments of Achilles tendon ruptures (ATRs) treated operatively or nonoperatively to guide clinical treatment choices.

**Methods:**

A literature search was performed in the PubMed, Cochrane Library, and Embase databases up to March 1, 2025, for randomized controlled trials (RCTs) involving patients with ATR receiving operative and nonoperative therapies. Primary outcomes included re-rupture rates, complications, and functional assessment. Meta-analysis of the extracted data was carried out using Review Manager 5.3 and Stata 17.0.

**Results:**

A total of 14 RCTs were included in the meta-analysis, comprising 1,628 participants. The meta-analysis results revealed a considerably lower re-rupture rate in both the minimally invasive (MI) group (risk ratio [RR], 0.28; 95% confidence interval [CI]: 0.11 to 0.74) and the open group (RR, 0.30; 95% CI: 0.19 to 0.50). For complications, subgroup analysis showed no significant difference between the MI and nonoperative groups (RR, 2.40; 95% CI: 0.52 to 10.98), whereas the open group had a higher complication rate (RR, 3.03; 95% CI: 1.75 to 5.26) than nonoperative groups. There was no significant difference in the functional assessment between operative and nonoperative groups. Regarding return to work, the MI group returned to work earlier compared to the nonoperative group.

**Conclusion:**

Open operative treatment significantly reduces the rate of re-rupture compared to nonoperative treatment but is accompanied by a higher risk of complications. MI treatment offers both of these advantages, along with superiority in return to work.

## Introduction

The Achilles tendon is one of the thickest and largest tendons in the human body, playing an important role in walking, standing, running, jumping, and maintaining balance [1]. Achilles tendon rupture (ATR) is a common injury, with most ATRs caused by indirect trauma. The incidence of ATR is 7 − 40 instances per 100,000 per year and shows an increasing trend, with the majority of ATRs occurring in young to middle-aged males, with an average age of 37 − 44 years [2–4]. ATRs are diagnosed primarily by a palpable tendon gap and the Simmonds [5] or Thompson test [6]. The exact degree or location of the rupture can be determined by ultrasonography or magnetic resonance imaging [7].

The most appropriate treatment for ATR remains debatable. Compared to nonoperative treatment, operative treatment has been reported to reduce the rate of re-rupture but at the expense of a higher incidence of complications (e.g., infection, skin-related adverse events, deep vein thrombosis [DVT], and sural nerve injury) [8–11]. Recently, there has been progress in nonoperative treatment, and studies have demonstrated similar results for both treatments [2,12]. Recently, there has been progress in nonoperative treatment, and studies have demonstrated similar results for both treatments [13,14]. The most common rehabilitation technique for ATRs is immobilization in a cast and maintaining a non-weight-bearing status for the first several weeks following the injury. However, extended immobility can lead to calf muscle atrophy, joint stiffness, and gait difficulties. Consequently, more studies are now promoting weight-bearing rehabilitation exercises earlier [15–22], although this remains controversial. Based on this, minimally invasive (MI) techniques have been developed to reduce the risk of complications associated with open operative [23].

Several meta-analyses have compared operative repair and nonoperative treatment of ATR patients [8,24,25], but these often have limitations, such as a small number of retrieved articles, lack of clarity in the analyses, or varying quality of included research. Furthermore, none of these studies have isolated MI treatment as an independent variable from operative treatment for dedicated analysis. Recent high-quality studies provide a greater number of experiments and updated evidence on this topic [20–22,26]. The purpose of this meta-analysis was to compile the most comprehensive set of randomized controlled trials (RCTs) currently available to assess re-rupture rates, complications, and functional outcomes after MI, open, and nonoperative treatments for ATR to assist in decision-making about ATR treatment.

## Methods

### Search strategy and trial selection

The Preferred Reporting Items for Systematic Reviews and Meta-Analyses (PRISMA) 2020 statement [27] and AMSTAR (Assessing the Methodological Quality of Systematic Reviews) Guidelines [28] were followed when conducting this systematic review and meta-analysis. The protocol for this systematic review is registered on PROSPERO (CRD42023460481). The PubMed/Medline, Cochrane Library, and Embase databases were systematically searched. Related publications up to March 1, 2025, were included in the initial screening, which was performed using a search strategy combining terms ((Achilles tendon[Title/Abstract]) OR (calcaneal tendon[Title/Abstract])) AND ((((((operative[Title/Abstract]) OR (surgical[Title/Abstract])) OR (repair[Title/Abstract])) OR (nonoperative[Title/Abstract])) OR (non-surgical[Title/Abstract])) OR (conservative[Title/Abstract])). The references in the included articles were further reviewed to identify additional studies. The inclusion and exclusion criteria are detailed in Table S1. To confirm that the selected publications matched the inclusion criteria, the titles and abstracts of the studies were blindly examined by two authors (SHX and YL). Disagreements over trial inclusion or data were resolved through discussion and consensus, with the help of a senior reviewer (JLX).

### Data extraction, synthesis, and assessment of the outcomes

The extracted data included the following: study period, country, study design, level of evidence, sex, age, inclusion/exclusion criteria, operative and nonoperative techniques, side, follow-up duration, weight-bearing time, re-rupture rates, complications, and functional outcomes. The selected clinical outcomes were based on the most commonly used measures in recent publications. Additionally, we collected more comprehensive data, such as adverse events and functional assessments, including the Achilles Tendon Rupture Score (ATRS), Short Musculoskeletal Function Assessment (SMFA) dysfunction score, Leppilahti Score, return to work, ankle range of motion, and calf atrophy. For adverse events and functional outcomes, the primary adverse event was re-rupture. Secondary adverse events included four complications: DVT, skin-related complications, deep wound infection, and sural nerve lesions. Skin-related complications included small skin openings, pressure sores, scars or skin adhesions, blisters, and superficial wound infections. Functional outcomes encompassed functional scores, return to work time, calf atrophy, and ankle range of motion. Two reviewers (SHX and EBL) independently retrieved the relevant data from the included studies and entered it into a spreadsheet for easy reference (Excel 2021, Microsoft), which was then reviewed by two senior reviewers (JLX and XYS).

### Study quality assessment and risk of bias assessment

The Cochrane Collaboration’s risk of bias assessment tool was used to evaluate the methodological quality of the RCTs. Two reviewers (SHX and XYS) independently assessed the included RCTs for risk of bias. Any disagreements between the reviewers were discussed and resolved through consensus.

### Statistical analysis

Meta-analysis of the extracted data was conducted using Review Manager 5.3 and Stata 17.0. Methods outlined in the *Cochrane Handbook for Systematic Reviews of Interventions* were used to convert continuous variables into mean and standard deviation values [29]. Dichotomous variables were extracted as absolute numbers and percentages, evaluated using the Mantel-Haenszel method, and expressed as risk ratios (RRs) with 95% confidence intervals (CIs). A random-effects model was applied if there was high heterogeneity (defined as I^2^> 50%) between studies; otherwise, a fixed-effects model was used [30]. Potential publication bias was assessed through funnel plots using the RR and standard error [31]. To evaluate the outcomes of operative versus nonoperative treatment across different follow-up durations, data for all follow-up time points were recorded. If relevant outcomes were reported at multiple follow-up points, the data were analyzed separately for each point. All eligible studies were included in the meta-analyses and subgroup analyses, as applicable. *p* < 0.05 was considered statistically significant.

## Results

### Search results and studies included

**Figure 1.**
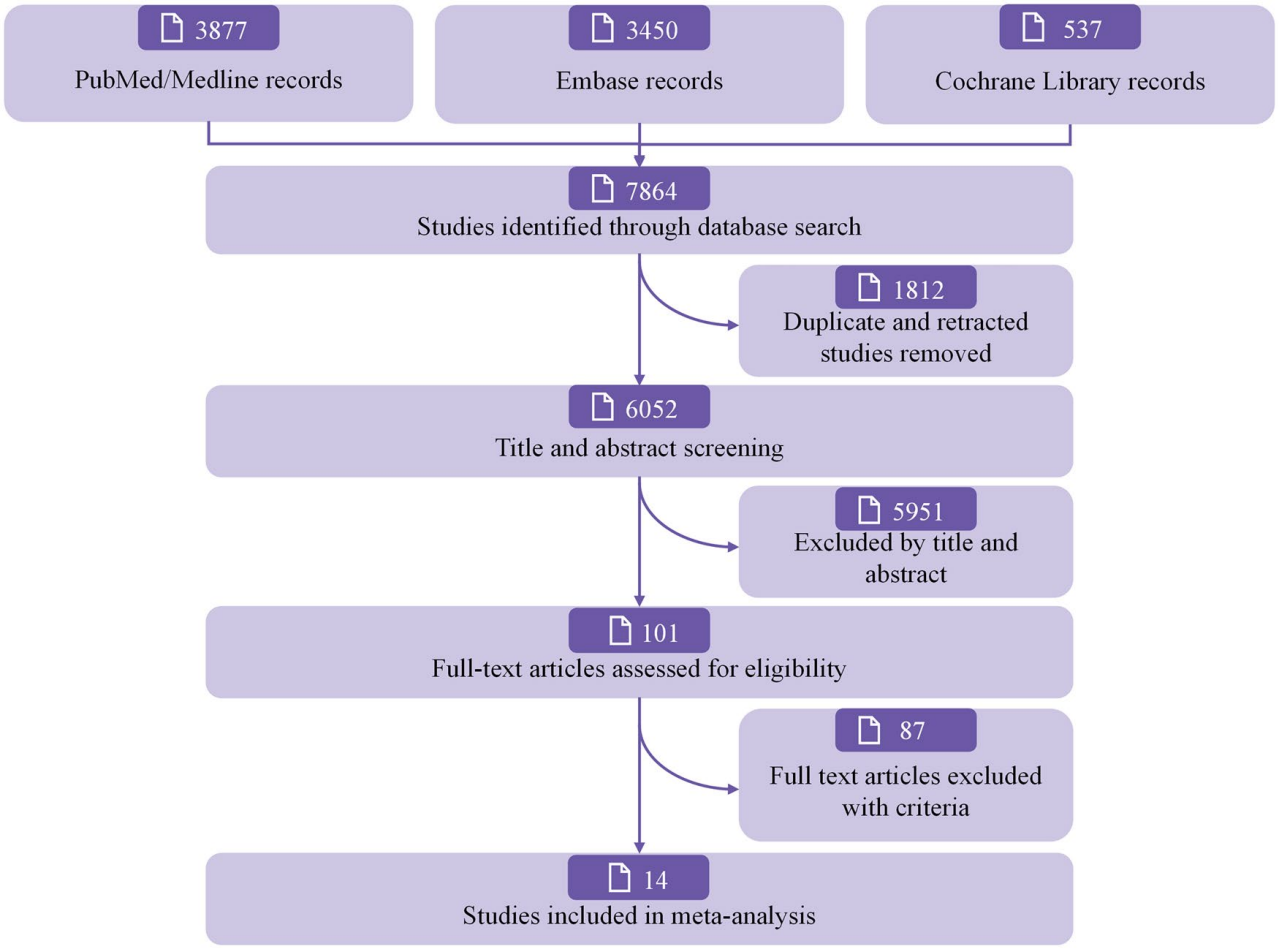
PRISMA Flowchart showing the selection process for the included randomized clinical trials.

Figure 1 shows the flowchart for the literature search and study selection. The electronic search of the aforementioned databases identified 5,945 relevant studies. After removing duplicates, 3,706 articles were screened based on their titles and abstracts. Seventy-five studies were deemed relevant, and their full texts were reviewed for eligibility. Finally, 14 RCTs [16–22,26,32–37] were considered eligible and included in the quantitative analysis.

### Risk of bias

**Figure 2.**
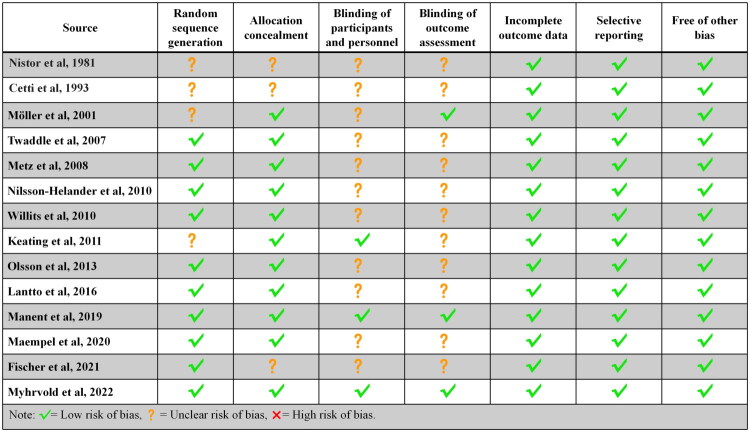
Risk of bias of assessment for the included randomized clinical trials using Cochrane Collaboration’s tool.

The Cochrane risk of bias tool includes seven items: randomization generation, allocation concealment, participant and personnel blinding, outcome assessment blinding, incomplete outcome data, selective reporting, and other biases. All included studies were assessed for risk of bias and categorized as low, unclear, or high risk. The evaluation results indicated that all studies had a lower risk of bias (Figure 2).

### Baseline characteristics

The baseline characteristics of the 14 RCTs included in this meta-analysis are shown in Table 1. The overall number of participants was 1,628; of these, 903 patients were treated operatively (MI: 255; open: 648) and 725 nonoperatively. Among the participants, 1,327 (80%) were male.

**Table 1.** Baseline characteristics of the included studies.

Source	Study period	Country	Study design	LOE	Overall number of patients	Number		Mean (SD or range) age, years		Sex (female/male)		Side (left/right)		Time between injury and treatment (days)	Follow-up, months (mean ± SD)
						OP	NON	OP	NON	OP	NON	OP	NON		
Nistor [32]	1973-1977	Sweden	RCT	NA	107	46	61	41 ± 9.3		11/96		NA	NA	NA	30 ± 8
Cetti et al. [33]	1982-1984	Denmark	RCT	NA	111	56	55	37.2 (21–62)	37.8 (21–65)	9/47	10/45	35/21	30/25	OP: 0.7 (0–9) NON: 0.6 (0–7)	12
Möller et al. [34]	1995-1997	Sweden	RCT	NA	112	59	53	39.6 (21–63)	38.5 (26–59)	8/51	5/48	34/25	30/23	7 days or less	24
Twaddle and Poon [35]	1997-2002	New Zealand	RCT	I	50	25	25	41.8	40.3	6/14*	8/14*	10/10*	12/10*	2 days or less	12
Metz et al. [16]	2004-2005	Netherlands	RCT	II	83	42	41	40 (23–63)	41 (25–62)	11/31	6/35	28/14	21/20	3 days or less	12
Nilsson-Helander et al. [36]	2004-2007	Sweden	RCT	I	97	49	48	40.9 ± 8.8	41.2 ± 9.5	9/40	9/39	26/23	21/27	3 days or less	12
Willits et al. [17]	2000-2005	Canada	RCT	I	144	72	72	39.7 ± 11	41.1 ± 8.0	13/59	13/59	NA	NA	14 days or less	24
Keating et al. [37]	2000-2004;	United Kingdom	RCT	NA	80	39	41	41.2 (27–59)	39.5 (21–58)	11/28	9/32	NA	NA	10 days or less	12
Olsson et al. [18]	2009-2010	Sweden	RCT	I	100	49	51	39.8 ± 8.9	39.5 ± 9.7	10/39	4/47	24/25	16/35	4 days or less	12
Lantto et al. [19]	2009-2013	Finland	RCT	I	60	32	28	40 (27–57)	39 (28–60)	2/30	3/25	NA	NA	7 days or less	18
Manent et al. [20]	2014-2017	Spain	RCT	II	34	23	11	MI: 41 (18-50)^&^; Open: 40.5 (28-51)^&^	42 (26-51)^&^	2/21	1/10	18/5	9/2	10 days or less	12
Maempel et al. [26]	2017-2018	United Kingdom	RCT	NA	64	33	31	56.0 (37–75)^#^	59.4 (46–77)^#^	11/22	8/23	NA	NA	10 days or less	188 ± 8.5
Fischer et al. [21]	2012-2015	Germany	RCT	I	90	60	30	MI: 39.3 ± 7.9; Open: 39.6 ± 7.3	45.2 ± 9.5	6/54	3/27	29/31	13/17	NA	NA
Myhrvold et al. [22]	2013-2018	Norway	RCT	NA	526	348	178	MI: 39.1 ± 8.4; Open: 39.9 ± 8.9	39.9 ± 8.1	93/255	42/136	174/174	87/91	8 days or less	12

LOE: level of evidence; SD: standard deviation; RCT: randomized controlled trial; NA: not available; NON: nonoperative treatment; OP: operative treatment; MI: minimally invasive.

* Ratio may not add up to the total number of patients owing to loss to follow-up.

^#^ Age at time of follow-up.

^&^ Median (range).

The treatment characteristics of the studies are listed in Table S2. Among the operative treatments, the Kessler suture technique was used in five studies, while the Bunnell and Krackow techniques were each used in four studies. For nonoperative treatments, twelve studies employed cast immobilization, and eight studies allowed weight-bearing in less than four weeks.

The numbers of re-ruptures, complications, functional scores, and other outcome measures after treatment for both groups are shown in Tables S3 and S4. Table S3 indicates that the operative group experienced significantly fewer re-ruptures than the nonoperative group, while the opposite was true for complications.

### Domain 1: major adverse events

#### Re-ruptures

**Figure 3.**
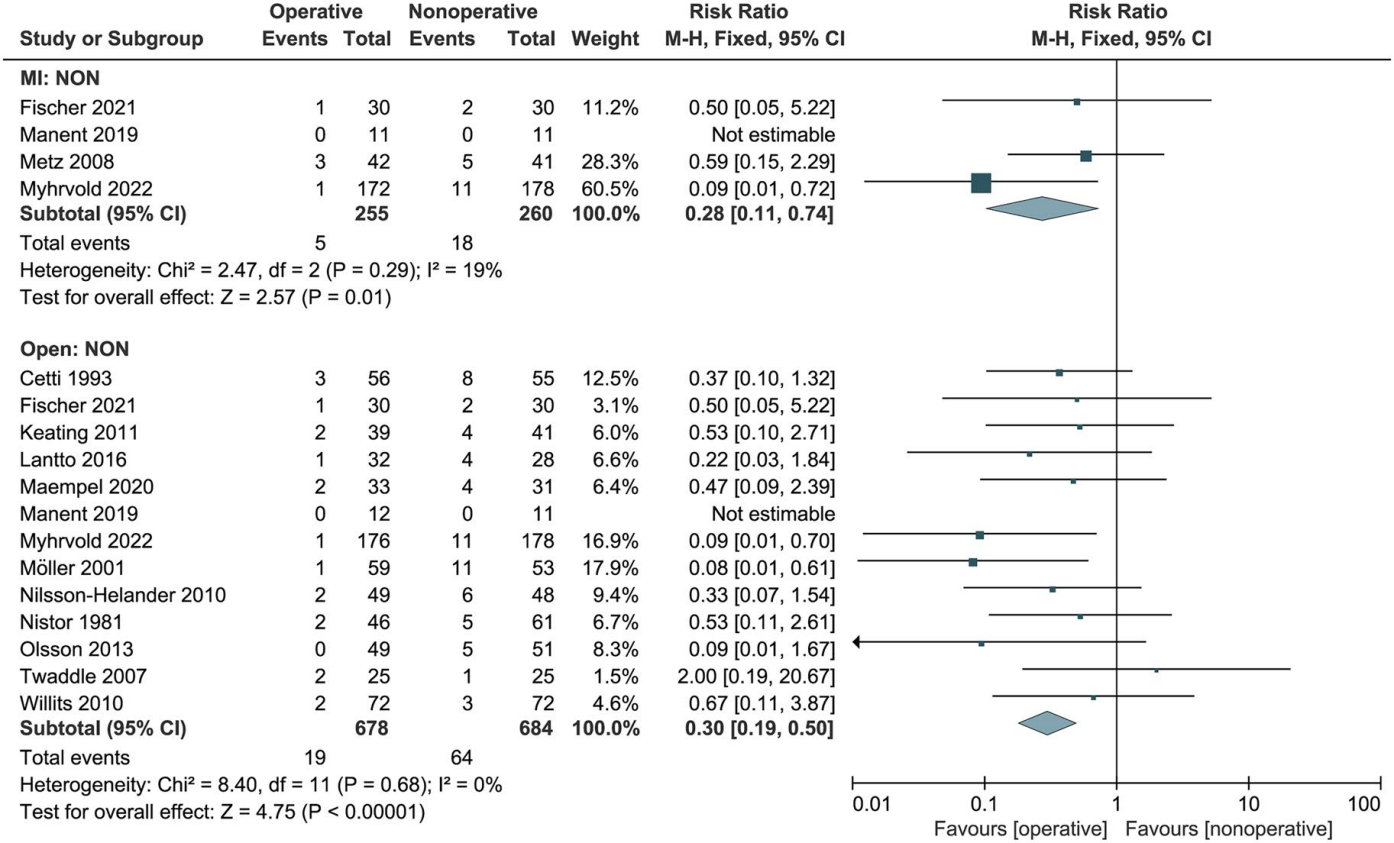
Forest Plot of re-rupture rate in a meta-analysis of Achilles tendon ruptures. NON: nonoperative treatment; MI: minimally invasive; M-H: Mantel-Haenszel; CI: confidence interval.

All studies reported on the occurrence of re-ruptures. Re-rupture occurred in 2.7% (24/903) of patients in the operative group and 9.5% (69/725) in the nonoperative group. Subgroup analysis revealed a considerably lower re-rupture rate in the MI and open groups (RR: 0.28, 95% CI: 0.11 to 0.74; RR: 0.33, 95% CI: 0.20 to 0.54) (Figure 3). There was no significant heterogeneity among the included studies (*p* = 0.70; I^2^ = 0%), so a fixed-effects model was used. The funnel plot showed no apparent asymmetry (Figure S1).

Eight studies [16–22,34] reported early weight-bearing within 4 weeks of treatment. Analysis showed fewer re-ruptures in the MI and open groups (RR: 0.28, 95% CI: 0.11 to 0.47; RR: 0.17, 95% CI: 0.08 to 0.39; I^2^ = 19%, 0%) (Figure S2A). Five studies [26,33,35–37] reported later weight-bearing, i.e., more than 4 weeks after initial treatment, and found that the open group also had a lower re-rupture rate (RR: 0.47, 95% CI: 0.24 to 0.94; *p* = 0.03; I^2^ = 0%) (Figure S2B).

### Domain 2: secondary adverse events

#### Complications

**Figure 4.**
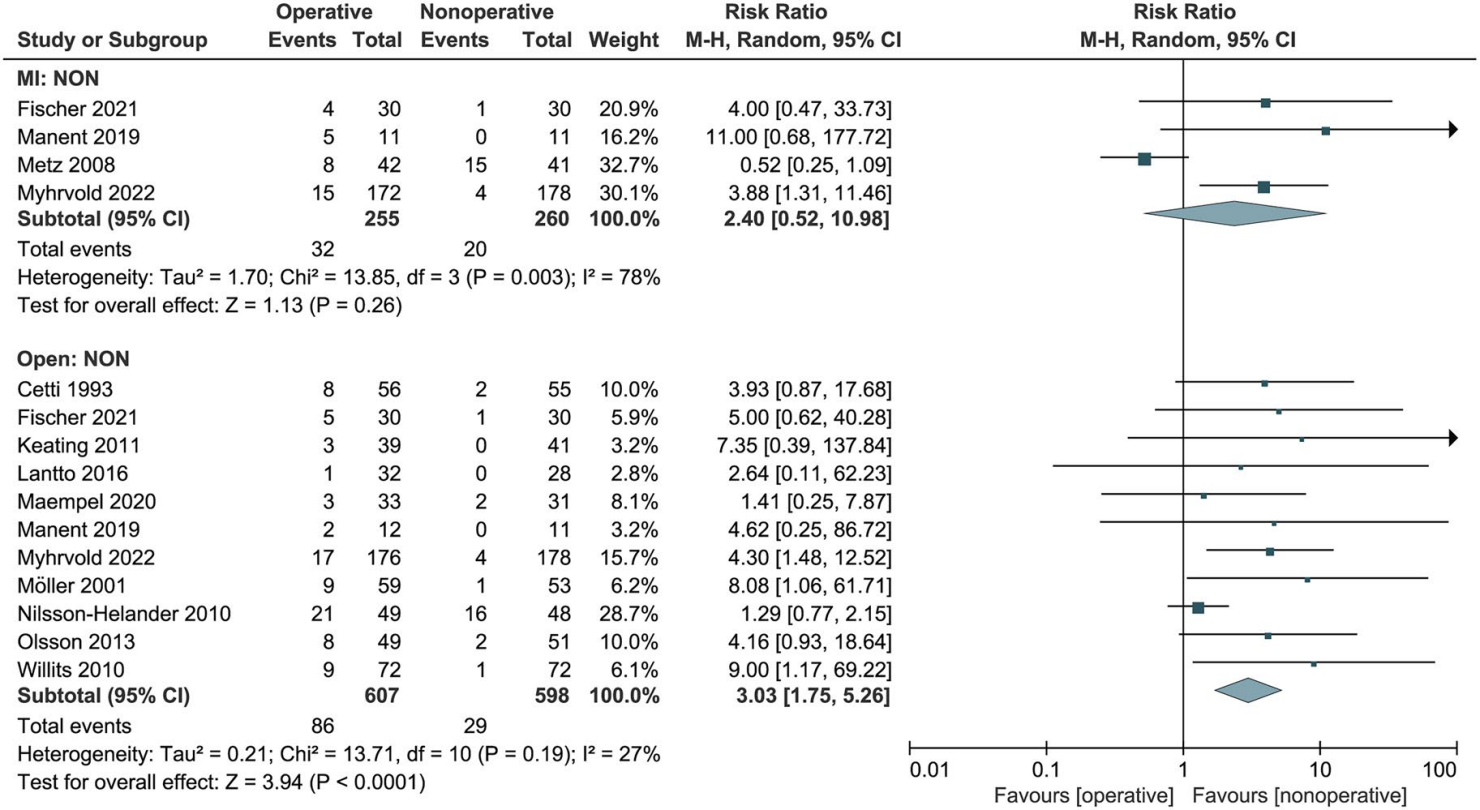
Forest Plot of complication rate in a meta-analysis of Achilles tendon ruptures. NON: nonoperative treatment; MI: minimally invasive; M-H: Mantel-Haenszel; CI: confidence interval.

Twelve studies [16–22,26,33,34,36,37] recorded complications. Subgroup analysis showed no significant difference between the MI and nonoperative groups (RR: 2.40, 95% CI: 0.52 to 10.98; *p* = 0.26). However, the open group had a higher complication rate (RR: 3.03, 95% CI: 1.75 to 5.26; *p* < 0.01) (Figure 4). A random-effects model was employed due to high heterogeneity (I^2^ = 78%) and moderate heterogeneity (27%).

We performed subgroup analyses for four complications. For skin-related adverse events (Figure S3), there was no statistical significance between the MI and nonoperative groups (RR: 1.15, 95% CI: 0.31 to 4.33; *p* = 0.84). However, the open group showed a higher incidence with significant differences (RR: 3.60, 95% CI: 1.81 to 7.13; *p* < 0.01). No heterogeneity was observed for the other three complications. Regarding DVT, no significant differences were observed between the open and nonoperative groups or between the MI and nonoperative groups (Figure S4). However, MI treatments were more likely to damage the sural nerve (RR: 5.86, 95% CI: 1.57 to 21.93; *p* < 0.01), whereas this was less frequent in the open group (RR: 4.03, 95% CI: 0.86 to 18.84; *p* = 0.08) (Figure S5). In contrast, the open group had a higher rate of deep wound infection compared to the nonoperative group (RR: 3.95, 95% CI: 1.13 to 13.81; *p* = 0.03), which was not observed in the MI group (RR: 7.24, 95% CI: 0.38 to 139.19; *p* = 0.19) (Figure S6).

### Domain 3: subjective functional outcomes

#### Achilles tendon rupture score

Five studies [18,20,22,26,36] reported the ATRS; of these, one study provided insufficient information [20], three reported short-term (≤ 1 year) scores [18,22,36], and one reported long-term scores [26]. ATRS was not reported in any study involving MI treatment. Subgroup analysis stratified by follow-up duration revealed that at 3 months, the operative group had significantly higher ATRS than the nonoperative group (MD: 4.40, 95% CI: 0.55 to 8.25; *p* = 0.02). However, during follow-up periods ranging from 0.5 to 15.7 years, no significant differences in ATRS were observed between the two groups (Figure S7).

#### SMFA dysfunction score

Two studies [26,37] reported SMFA dysfunction scores at 3 months to 1 year follow-up. Subgroup analysis stratified by follow-up duration showed that at 3 months, the operative group exhibited significantly higher SMFA dysfunction scores compared to the nonoperative group (MD: −4.53, 95% CI: −6.56 to −2.51; *p* < 0.01). However, no significant differences in SMFA dysfunction scores were observed between the two groups during the follow-up period from 4 to 12 months (Figure S8).

#### Leppilahti score

Two studies [17,19] reported Leppilahti scores. There was no significant difference in scores between the two groups at each follow-up point (Figure S9).

### Domain 4: objective functional outcomes

#### Return to work

Five studies [16,32–34,37] reported the average time patients took to return to work for the two groups; however, one study [37] was excluded due to inadequate reporting of information. Among the remaining four studies, heterogeneity was observed, and a random-effects model was applied. Subgroup analyses showed that MI treatment led to an earlier return to work compared to nonoperative treatment (MD: −7.00, 95% CI: −13.10 to −0.90; *p* = 0.02), while open operative treatment did not offer this advantage (MD: −0.34, 95% CI: −4.57 to 3.89, *p* = 0.88) (Figure S10).

#### Ankle range of motion (ROM)

**Figure 5.**
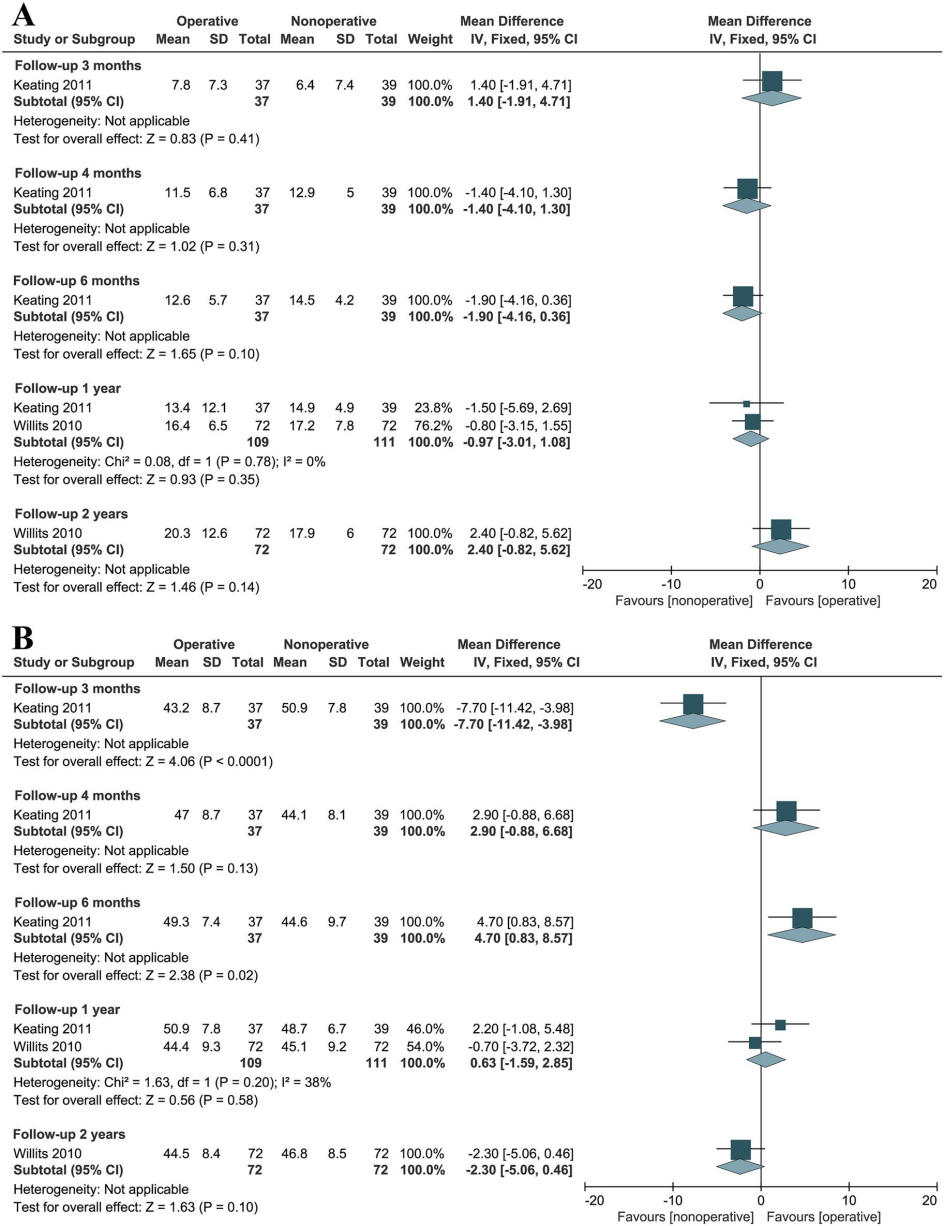
Forest Plot of ankle dorsiflexion (A) and plantar flexion (B) in a meta-analysis of Achilles tendon ruptures. SD: standard deviation; CI: confidence interval.

Five studies [17,20,34,35,37] reported ankle ROM data. However, three studies were excluded due to insufficient information, leaving only two studies for analysis [17,37]. ROM was not reported in any study involving MI treatment. Over a follow-up period ranging from 0.25 to 2 years, no significant differences were observed between the two groups in ankle dorsiflexion (Figure 5A). Regarding plantarflexion, there were no significant differences between the operative and nonoperative groups at 4 months, 1 year, and 2 years of follow-up (Figure 5B). However, at 3 months follow-up, the nonoperative group demonstrated significantly greater plantarflexion than the operative group (MD: −7.70, 95% CI: −11.42 to −3.98; *p* < 0.01). Conversely, at 6 months of follow-up, plantarflexion was significantly greater in the operative group compared to the nonoperative group (MD: 4.70, 95% CI: 0.83 to 8.57; *p* = 0.02) (Figure 5B).

#### Calf atrophy

**Figure 6.**
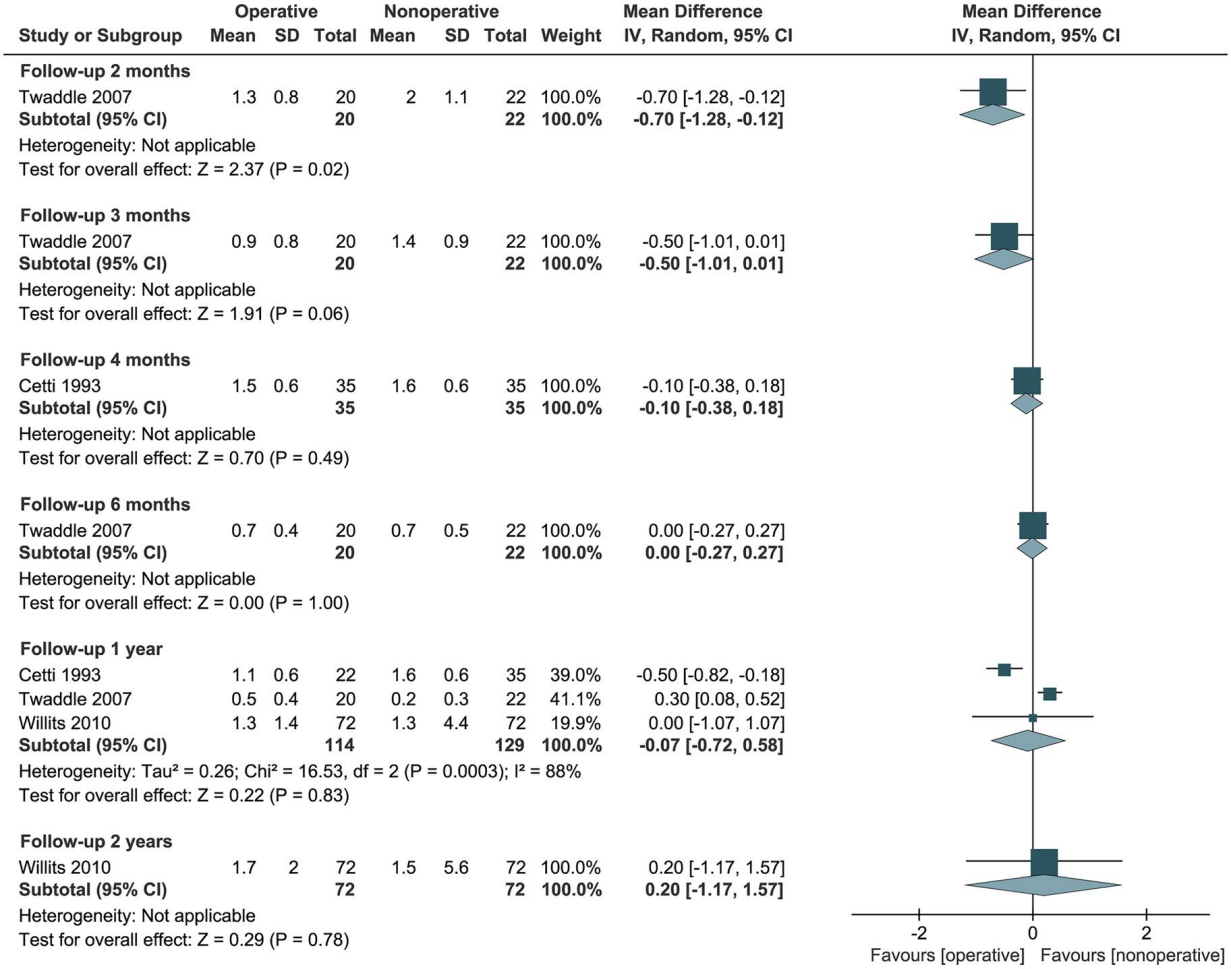
Forest Plot of calf atrophy in a meta-analysis of Achilles tendon ruptures. SD: standard deviation; CI: confidence interval.

Five studies [17,20,32,33,35] reported calf atrophy, but two studies [20,32] were excluded due to inadequate reporting of information. For the remaining three RCTs, calf atrophy was more severe in the nonoperative group at 2 months of follow-up (MD: −0.70, 95% CI: −1.28 to −0.12; *p* = 0.02). However, no significant differences were observed at 3 months (MD: −0.50, 95% CI: −1.01 to 0.01), 4 months (MD: −0.10, 95% CI: −0.38 to 0.18), 6 months (MD: 0.00, 95% CI: −0.27 to 0.27), 12 months (MD: −0.07, 95% CI: −0.72 to 0.58), and 24 months (MD: 0.20, 95% CI: −1.17 to 1.57) of follow-up (Figure 6).

## Discussion

To the best of our knowledge, this study represents the most comprehensive and detailed analysis of available RCTs on this topic to date. Evidence was synthesized from 14 RCTs, identified through a thorough search and screening process of major scientific literature databases, comparing re-ruptures, complications, and functional scores in patients with ATRs who underwent operative (MI and open) or nonoperative treatment. Our overall aim was to assist in decision-making regarding the most suitable therapy for ATR.

### Main findings

In domain 1, re-rupture occurred in 2.7% (24/903) of patients in the operative group (MI and open) and 9.5% (69/725) in the nonoperative group. Regarding early versus late weight-bearing, our findings indicated that for early weight-bearing rehabilitation (within 4 weeks of treatment), the re-rupture rate was 1.6% (11/685) in the operative group and 8.8% (41/464) in the nonoperative group. For late weight-bearing rehabilitation (more than 4 weeks after treatment), the re-rupture rate was 5.4% (11/202) in the operative group and 11.5% (23/200) in the nonoperative group, with an RR of 0.44 between the two groups. According to these statistics, both MI and open operative treatments were associated with a decreased risk of re-rupture compared to the nonoperative group.

In domain 2, 12 studies reported complications other than re-rupture. Patients receiving nonoperative treatment experienced fewer complications compared to those receiving open operative treatment, but no significant difference was observed between nonoperative and MI treatments. Specifically, the operative group had a higher frequency of skin-related adverse events and deep wound infections than the nonoperative group, while MI treatment was associated with a higher incidence of sural nerve lesions compared to nonoperative treatment.

In domain 3, the subjective functional outcomes analyzed were the ATRS, SMFA dysfunction score, and Leppilahti score. None of the functional scores involved MI treatment. Our study analyzed these three functional assessments at various follow-up time points. The analysis revealed no significant differences in ATRS and Leppilahti scores between the two groups, but the operative group demonstrated superior outcomes compared to the nonoperative group in terms of the SMFA dysfunction score.

In domain 4, the objective functional outcomes assessed included the time to return to work, calf atrophy, and ROM. Our meta-analysis found that MI treatment was associated with a significantly quicker return to work (MD: −7.00, 95% CI: −13.10 to −0.90; *p* = 0.02). Subgroup analyses based on follow-up time indicated that the operative group experienced less calf atrophy at 2 months, while the nonoperative group had an advantage in ankle plantar flexion at 3 months. No significant differences were observed in the other outcomes.

### Feasibility interpretation

The incidence of ATR continues to rise, although the reasons for this increase remain unclear. Major risk factors include aging (particularly individuals over 60 years old), obesity, inflammatory diseases, and a history of oral quinolone or corticosteroid use [38]. Re-rupture of the Achilles tendon imposes significant psychological and economic burdens on patients, and so avoiding re-rupture is a key requirement. One potential reason for the higher re-rupture rates associated with nonoperative treatment could be related to the tendon healing process. Tendons heal through both intrinsic and extrinsic mechanisms. Intrinsic repair involves the participation of inflammatory cells and fibroblasts within the tendon [39], while extrinsic healing relies on cell migration from the surrounding area, including the synovium and adjacent tendon sheath [39,40]. In nonoperative treatment, extrinsic healing predominates, which may lead to a larger gap between the tendon ends compared to operative treatment. Internal healing, characteristic of operative repair, generally results in more favorable biomechanical conditions [40]. Extrinsic healing can also lead to increased adhesion and scar tissue formation, which may impair the tendon’s normal sliding function [40]. Tendons repaired surgically exhibit better biomechanical properties, potentially explaining the lower re-rupture rate compared to nonoperative approaches.

### Comparison with other studies

Compared to nonoperative treatment, both open and MI operative techniques exhibit a lower risk of re-rupture. These findings are consistent with those reported in other meta-analyses [8,25,41]. However, Van der Eng et al. [42] found no significant difference in re-rupture rates between operative and nonoperative treatments. This discrepancy may be attributed to potential biases in data analysis or the limited number of studies included in their review.

Among the four types of complications, the MI group showed a higher incidence only of sural nerve lesions. In contrast, the open operative group had a significantly higher incidence of skin-related adverse events and deep wound infections compared to the nonoperative group. Zhou et al. [43] reported that the operative group had significantly higher rates of deep wound infections, adhesions, and sural nerve lesions compared to the nonoperative group. Conversely, Reda et al. [25] found differences only in superficial infections and reported no statistically significant differences for DVT, deep wound infections, or sural nerve lesions. These results differ from ours, likely due to Zhou and Reda combining MI and open treatments into a single category, which may obscure the advantages of MI techniques. Systematic reviews by Rozis et al. [44] and Yang et al. [45] found lower complication rates and better functional outcomes with MI treatment compared to open surgery.

In this study, MI intervention was associated with a shorter recovery period for patients returning to work. Soroceanu et al.’s [11] meta-analysis found that patients who underwent operative treatment returned to work an average of 19.16 days earlier than those who received nonoperative treatment. Conversely, Ochen et al. [8] who analyzed both randomized controlled trials and observational studies, reported no significant difference in return-to-work times between the operative and nonoperative groups. Given the limited data on MI treatment, further research is needed to explore differences in return-to-work times among MI, open, and nonoperative treatments.

### Strengths and limitations

Our study has several advantages. To our knowledge, it includes the largest pool of RCTs to date. We conducted a thorough analysis of functional outcomes using multiple evaluation metrics at various follow-up time points. Unlike previous studies that combined different operative treatments, we differentiated between open and MI treatments and performed several subgroup analyses stratified by follow-up time to ensure the robustness of our findings.

Similarly, there are some limitations of our research efforts to mention. First, results may be affected by missing articles. Second, different measurements may have been used on the same item between different studies, which could have affected the results of the studies. Finally, different treatment techniques and rehabilitation programs may affect the results of each study.

## Conclusions

Our systematic review and meta-analysis of RCTs revealed that, compared with nonoperative treatment, open operative treatment significantly reduces the rate of re-rupture but is associated with a higher risk of complications. MI treatment offers the benefits of reduced re-rupture rates and superiority for return to work, demonstrating broad application potential. However, further high-quality RCTs are needed to better evaluate its superiority over nonoperative treatment.

## Supplementary Material

PRISMA CHECKLIST.docx

Supplementary materials.docx

## Data Availability

All data are secondary and are available in the original published studies. Data produced through meta-analysis will be available with publication (in the manuscript and/or Supplementary Material). Additional information can be supplied by the corresponding author upon reasonable request.
