# Supplementary material for: Operative versus nonoperative management of acute Achilles tendon rupture: a systematic review and meta-analysis of clinical outcomes from randomized controlled trials
Source: Ann Med. 2025 Nov 17;57(1):2537349. doi: 10.1080/07853890.2025.2537349 (PMC12624965; doi:10.1080/07853890.2025.2537349)
Supplement: Supplementary materials.docx [file IANN_A_2537349_SM6848.docx]

**Supplemental Online Content**

**Supplementary Table 1** Study inclusion and exclusion criteria

**Supplementary Table 2** Treatment characteristics of the included studies

**Supplementary Table 3** Post-treatment functional scores of the included studies

**Supplementary Table 4** Other outcome measures of the included studies

**Supplementary Figure 1** Funnel plot of re-rupture rate in a meta-analysis of Achilles tendon ruptures.

**Supplementary Figure 2** Forest plot of re-rupture rate with ≤ 4 weeks (A) and >4 weeks (B) weight-bearing in a meta-analysis of Achilles tendon ruptures.

**Supplementary Figure 3** Forest plot of skin-related adverse events rate in a meta-analysis of Achilles tendon ruptures.

**Supplementary Figure 4** Forest plot of deep vein thrombosis rate in a meta-analysis of Achilles tendon ruptures.

**Supplementary Figure 5** Forest plot of sural nerve lesions rate in a meta-analysis of Achilles tendon ruptures.

**Supplementary Figure 6** Forest plot of deep wound infection rate in a meta-analysis of Achilles tendon ruptures.

**Supplementary Figure 7** Forest plot of Achilles tendon rupture scores in a meta-analysis of Achilles tendon ruptures.

**Supplementary Figure 8** Forest plot of short musculoskeletal function assessment dysfunction score in a meta-analysis of Achilles tendon ruptures.

**Supplementary Figure 9** Forest plot of Leppilahti scores in a meta-analysis of Achilles tendon ruptures.

**Supplementary Figure 10** Forest plot of return to work in weeks in a meta-analysis of Achilles tendon ruptures.

**Supplementary Table 1** Study inclusion and exclusion criteria

| **Inclusion Criteria** | **Exclusion Criteria** |
| --- | --- |
| Achilles tendon rupture | Non-English articles |
| Randomized clinical trial | Case reports, case series, and reviews |
| Operative treatment (open or minimally invasive surgery) versus nonoperative treatment | Not comparing clinical outcomes between operative and nonoperative treatment |
| Age 18 years old or older | Unable to get full text information |
| Reported re-rupture rates, complication rates, or functional results | Reported on the same patient cohorts |

**Supplementary Table 2** Treatment characteristics of the included studies.

| **Source** | **Overall**  **number** | **Number** | | **Operative method** | **Operative suture technique** | **Nonoperative treatment** | **Weight-bearing ≤ 4 weeks** |
| --- | --- | --- | --- | --- | --- | --- | --- |
|  |  | **OP** | **NON** |  |  |  |  |
| Nistor et al., 1981 | 107 | 46 | 61 | Open | Bunnell | Cast | NA |
| Cetti et al., 1993 | 111 | 56 | 55 | Open | Bunnell | Cast | No |
| Möller et al., 2001 | 112 | 59 | 53 | Open | Kessler | Cast | Yes |
| Twaddle et al., 2007 | 50 | 25 | 25 | Open | Krackow | Cast/orthosis | No |
| Metz et al., 2008 | 83 | 42 | 41 | MI | Bunnell | Cast/brace | Yes |
| Nilsson-Helander et al., 2010 | 91 | 49 | 48 | Open | Kessler | Cast/brace | No |
| Willits et al., 2010 | 144 | 72 | 72 | Open | Krackow | Brace | Yes |
| Keating et al., 2011 | 80 | 39 | 41 | Open | Kessler | Cast | No |
| Olsson et al., 2013 | 100 | 49 | 51 | Open | Kessler | Brace | Yes |
| Lantto et al., 2016 | 60 | 32 | 28 | Open | Krackow | Cast/orthosis | Yes |
| Manent et al., 2019 | 34 | 23 | 11 | MI; Open | MI: Ma-Griffith Open: Bunnell | Cast | Yes |
| Maempel et al., 2020 | 64 | 33 | 31 | Open | Kessler | Cast | No |
| Fischer et al., 2021 | 90 | 60 | 30 | MI; Open | NA | Cast | Yes |
| Myhrvold et al., 2022 | 526 | 348 | 178 | MI; Open | MI: Dresden Open: Krackow | Cast/orthosis | Yes |

NA, not available; NON, nonoperative treatment; OP, operative treatment; MI, minimally invasive.

**Supplementary Table 3** Post-treatment functional scores of the included studies

| **Source** | **Re-rupture**  **(n)** | | **Complication (n)** | | **ATRS**  **(mean, SD)** | | **Leppiahti score**  **(mean, SD)** | | **SMFA dysfunction score**  **(mean, SD)** | | **PAS**  **(mean, SD)** | |
| --- | --- | --- | --- | --- | --- | --- | --- | --- | --- | --- | --- | --- |
|  | **OP** | **NON** | **OP** | **NON** | **OP** | **NON** | **OP** | **NON** | **OP** | **NON** | **OP** | **NON** |
| Nistor et al., 1981 | 2 | 5 | NA | NA | NA | NA | NA | NA | NA | NA | NA | NA |
| Cetti et al., 1993 | 3 | 8 | 8 | 2 | NA | NA | NA | NA | NA | NA | NA | NA |
| Möller et al., 2001 | 1 | 11 | 9 | 1 | NA | NA | NA | NA | NA | NA | NA | NA |
| Twaddle et al., 2007 | 2 | 1 | 0 | 0 | NA | NA | NA | NA | NA | NA | NA | NA |
| Metz et al., 2008 | 3 | 5 | 8 | 15 | NA | NA | 26 of patients had good or excellent scores | 24 of patients had good or excellent scores | NA | NA | NA | NA |
| Nilsson-Helander et al., 2010 | 2 | 6 | 21 | 16 | 6 months: 72±17.3;  12 months: 88±17.5 | 6 months: 71±17;  12 months: 86±17.3 | NA | NA | NA | NA | 6 months: 3.4±1.3;  12 months: 3.6±1.3 | 6 months: 3.3±1.0;  12 months: 3.7±1.0 |
| Willits et al., 2010 | 2 | 3 | 9 | 1 | NA | NA | 12 months: 78.5±10.9;  24 months: 82.6±11.1 | 12 months: 76.3±15.8;  24 months: 82.2±12.3 | NA | NA | NA | NA |
| Keating et al., 2011 | 2 | 4 | 3 | 0 | NA | NA | NA | NA | 3 months: 15±0.5;  4 months: 8±0.8;  6 months: 3.9±0.7;  12 months: 1.2±0.3 | 3 months: 20±1.0;  4 months: 10±1.3;  6 months: 4.7±0.9;  12 months: 1.9±0.4 | NA | NA |
| Olsson et al., 2013 | 0 | 5 | 8 | 2 | 3 months: 43±20;  6 months: 70±23;  12 months: 82±20 | 3 months: 35±14;  6 months: 70±19;  12 months: 80±23 | NA | NA | NA | NA | 6 months: 3.8±1.0;  12 months: 4.0±1.1 | 6 months: 3.8±1.1;  12 months: 4.0±1.0 |
| Lantto et al., 2016 | 1 | 4 | 1 | 0 | NA | NA | 18 months: 79.5±10.3 | 18 months: 75.7±11.2 | NA | NA | NA | NA |
| Manent et al., 2019 | 0 | 0 | 7 | 0 | NA | NA | NA | NA | NA | NA | NA | NA |
| Maempel et al., 2020 | 2 | 4 | 3 | 2 | Mean follow-up of 15.7 years: 93.3±10.9 | Mean follow-up of 15.7 years: 91.8±14.8 | NA | NA | 3 months: 15.6±5.6;  4 months: 7.9±5.7;  6 months: 2.9±2.2;  12 months: 0.5±1.2 | 3 months: 17.9±12.7;  4 months: 9.6±7.8;  6 months: 3.4±4.0;  12 months: 1.7±2.9 | NA | NA |
| Fischer et al., 2021 | 2 | 2 | 9 | 2 | NA | NA | NA | NA | NA | NA | NA | NA |
| Myhrvold et al., 2022 | 2 | 11 | 32 | 4 | MI: 3 months: 43.8±1.1;  6 months: 62.4±1.1;  12 months: 78.9±1.0.  Open: 3 months: 50.5±1.1;  6 months: 65.6±1.1;  12 months: 77.7±1.0 | 3 months: 7.8±1.1;  6 months: 64.8±1.0;  12 months: 76.6±1.0 | NA | NA | NA | NA | NA | NA |

ATRS, Achilles Tendon Rupture Score; SMFA, Short Musculoskeletal Function Assessment; PAS, physical activity score. NA, not available; NON, nonoperative treatment; OP, operative treatment; SD, standard deviation; w, week; n, number; MI, minimally invasive.

**Supplementary Table 4** Other outcome measures of the included studies

| **Source** | **Ankle range of motion**  **(°, dorsiflexion, mean, SD or range)** | | **Ankle range of motion**  **(°, plantarflexion, mean, SD or range)** | | **Calf atrophy**  **(cm, mean, SD or range)** | | **Return to work**  **(w, mean, SD)** | | |  |
| --- | --- | --- | --- | --- | --- | --- | --- | --- | --- | --- |
|  | **OP** | **NON** | **OP** | **NON** | **OP** | **NON** | **OP** | | **NON** |  |
| Nistor et al., 1981 | NA | NA | NA | NA | Reduced by as much as 4 cm, but most of the patients, had a reduction of 1 to 1.5 cm. | | 13±7.5 | | 9±11 |  |
| Cetti et al., 1993 | NA | NA | NA | NA | 4 months: 1.5 (0.5 to 3);  12 months: 1.1 (0.5 to 3) | 4 months:  1.6 (0.5 to 3);  12 months:  1.6 (0.5 to 3) | 6.2±4.6 | | 8±13 |  |
| Möller et al., 2001 | 12 months: increased by 42% | 12 months: increased by 48% | 12 months: reduced by 5.2±5.5 | 12 months: reduced by 4.1±4.6 | NA | NA | 8±7 | | 11±8 |  |
| Twaddle et al., 2007 | 2 months:  -12.9 (-3 to -31);  3 months:  -8.2 (0 to -23);  6 months:  -2.3 (1 to -10);  12 months:  -1 (2 to -6) | 2 months:  -13.8 (-2 to -35);  3 months:  -8.0 (0 to -22);  6 months:  -2.4 (1 to -15);  12 months:  -0.2 (1 to -5) | 2 months:  -14.1 (-4 to -22);  3 months:  -8.2 (0 to -14);  6 months:  -2.3 (0 to -10);  12 months:  -0.6 (0 to -8) | 2 months:  -11.1 (-0 to -28);  3 months:  -6.0 (0 to -21);  6 months:  -2.7 (3 to -15);  12 months:  -0.2 (3 to -12) | 2 months:  -1.3 (-4 to 1);  3 months:  -0.9 (-3 to 0);  6 months:  -0.7 (-1.6 to 0);  12 months:  -0.5 (-1.6 to 0) | 2 months:  -2.0 (-5 to -0.5);  3 months:  -1.4 (-3.5 to 0);  6 months:  -0.7 (-2.1 to 0);  12 months:  -0.2 (-1.3 to 0) | NA | | NA |  |
| Metz et al., 2008 | NA | NA | NA | NA | NA | NA | 8.4±12 | | 15.4±16 |  |
| Nilsson-Helander et al., 2010 | NA | NA | NA | NA | NA | NA | NA | | NA |  |
| Willits et al., 2010 | 12 months: 16.4±6.5;  24 months: 20.3±12.6 | 12 months: 17.2±7.8;  24 months:  17.9±6.0 | 12 months: 44.4±9.3;  24 months:  44.5±8.4 | 12 months: 45.1±9.2;  24 months:  46.8±8.5 | 12 months:  -1.3±1.4;  24 months:  -1.7±2.0 | 12 months:  -1.3±4.4;  24 months:  -1.5±5.6 | NA | | NA |  |
| Keating et al., 2011 | 3 months: 7.8±7.3;  4 months: 11.5±6.8;  6 months: 12.6±5.7;  12 months: 13.4±12.1 | 3 months: 6.4±7.4;  4 months: 12.9±5.0;  6 months: 14.5±4.2;  12 months: 14.9±4.9 | 3 months: 43.2±8.7;  4 months: 47.0±8.7;  6 months: 49.3±7.4;  12 months: 50.9±7.8 | 3 months: 40.4±6.5;  4 months: 44.1±8.1;  6 months: 44.6±9.7;  12 months: 48.7±6.7 | NA | NA | 12 | | |  |
| Olsson et al., 2013 | NA | NA | NA | NA | NA | NA | NA | NA | | |
| Lantto et al., 2016 | NA | NA | NA | NA | NA | NA | NA | NA | | |
| Manent et al., 2019 | NA | NA | NA | NA | NA | NA | NA | NA | | |
| Maempel et al., 2020 | NA | NA | NA | NA | NA | NA | NA | NA | | |
| Fischer et al., 2021 | NA | NA | NA | NA | NA | NA | 4.5 | | |  |
| Myhrvold et al., 2022 | NA | NA | NA | NA | NA | NA | NA | NA | | |

cm, centimetre; SD, standard deviation; NA, not available; NON, nonoperative treatment; OP, operative treatment.

Negative values indicate the difference between the injured side and the non-injured side.


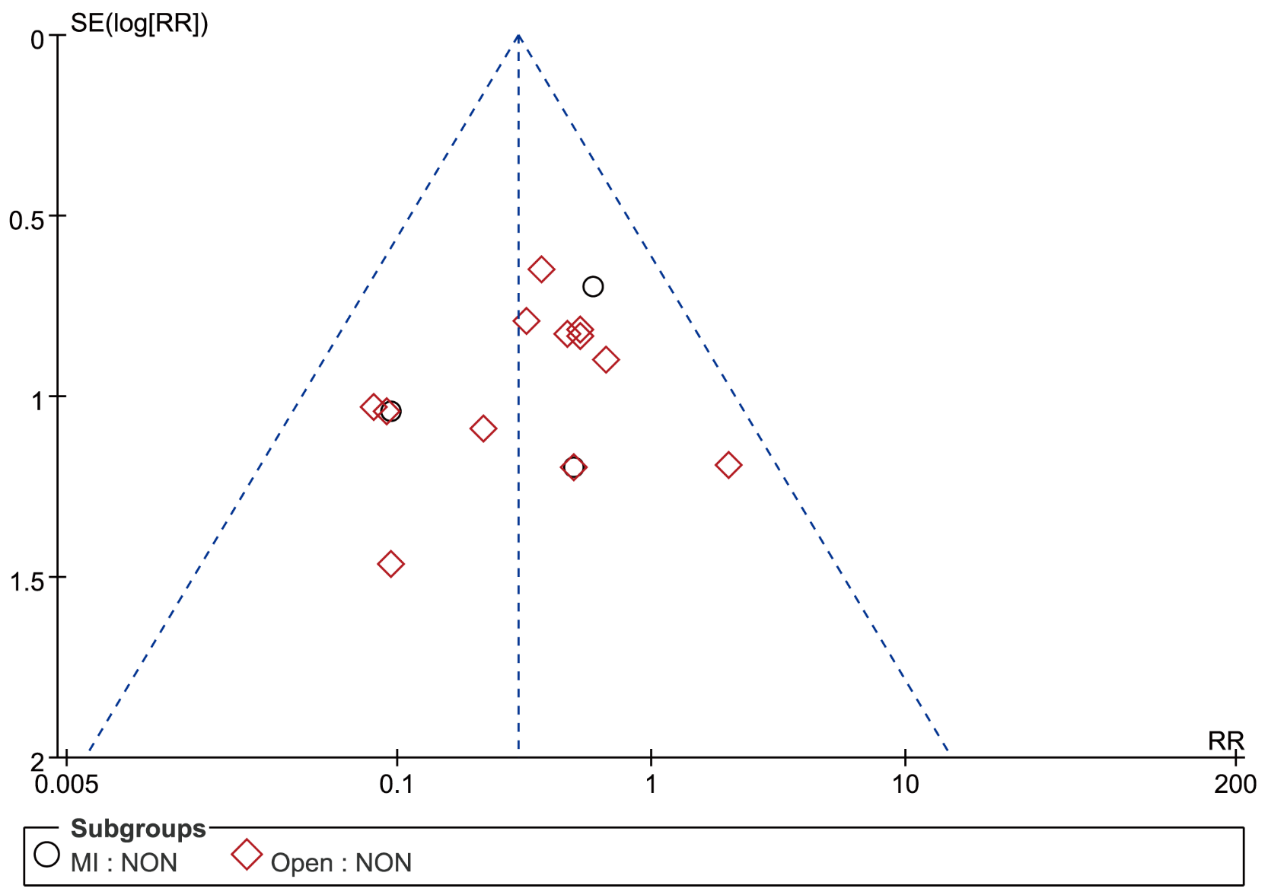


**Supplementary Figure 1** Funnel plot of re-rupture rate in a meta-analysis of Achilles tendon ruptures.

RR risk ratio; SE standard error; NON, nonoperative treatment; MI, minimally invasive.


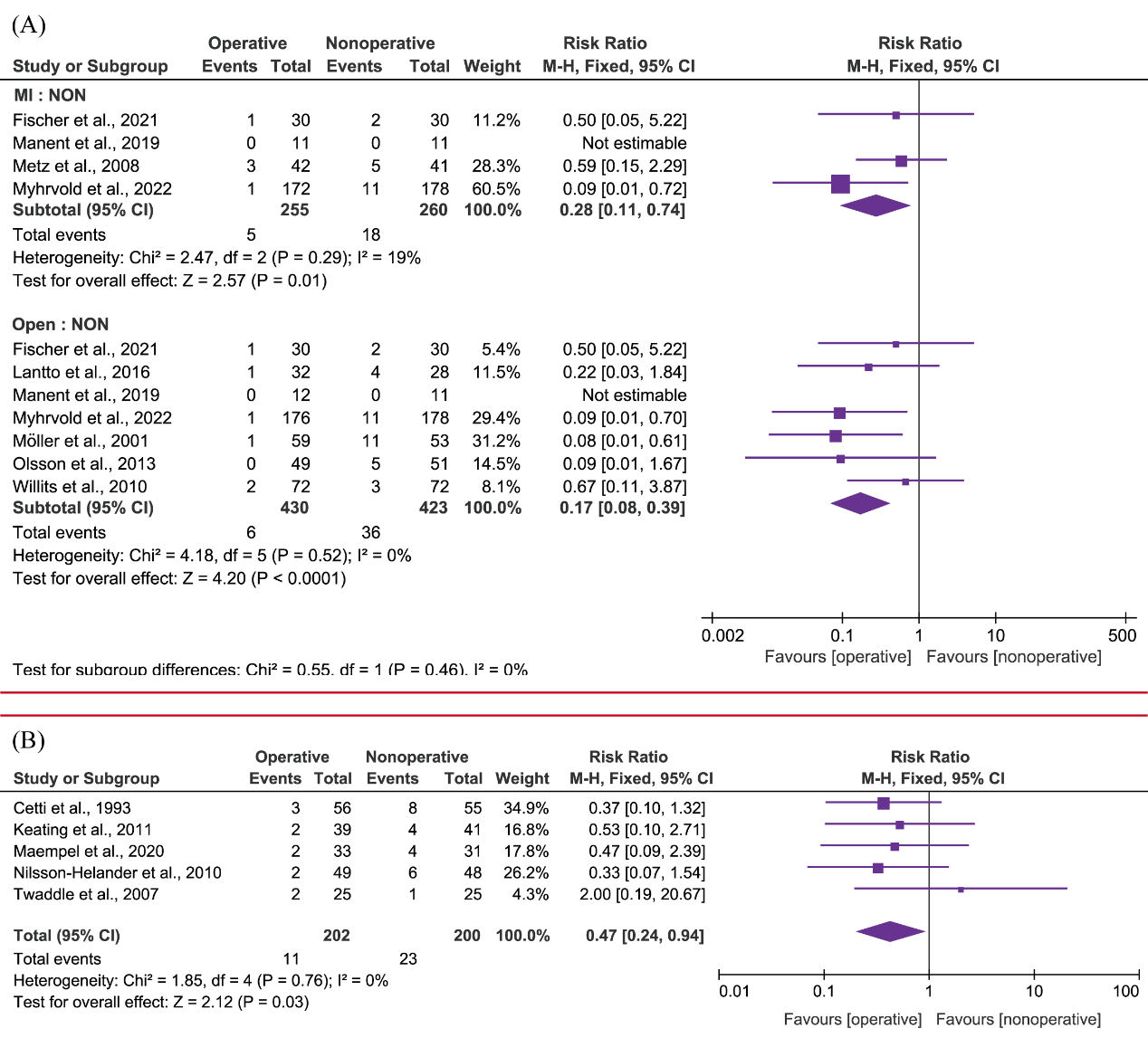


**Supplementary Figure 2** Forest plot of re-rupture rate with ≤ 4 weeks (A) and > 4 weeks (B) weight-bearing in a meta-analysis of Achilles tendon ruptures.

NON, nonoperative treatment; MI, minimally invasive; M-H, Mantel-Haenszel; CI, confidence interval.


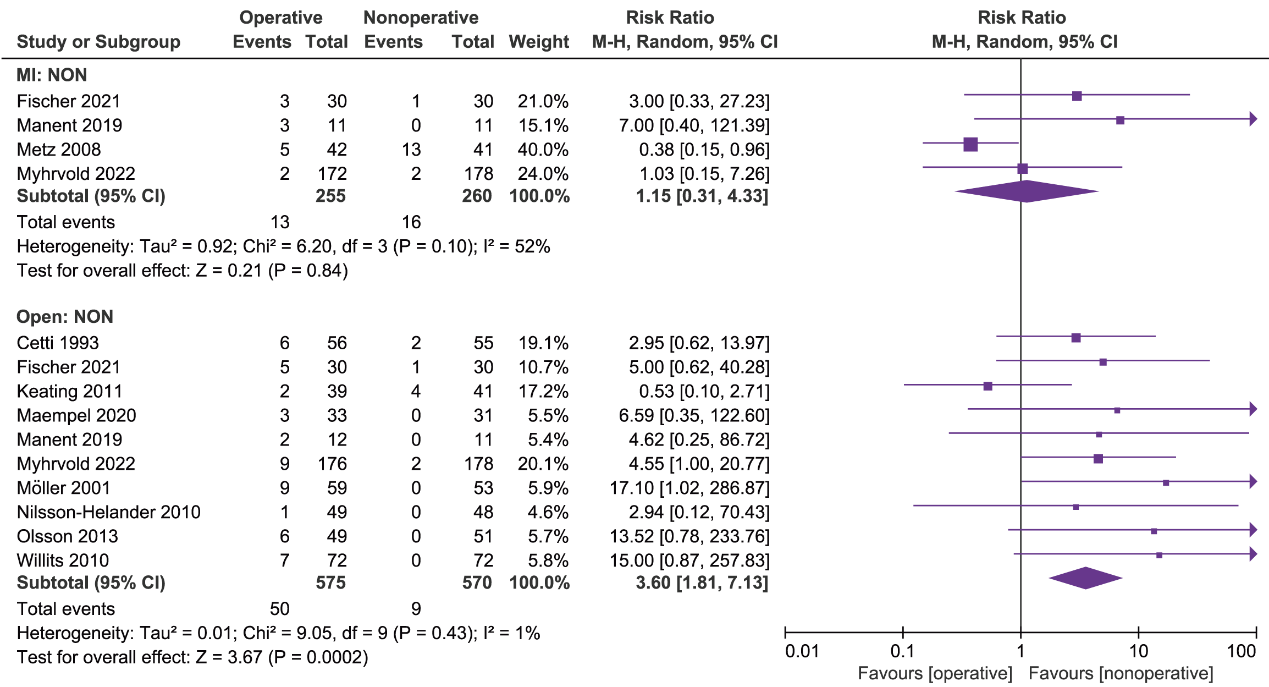


**Supplementary Figure 3** Forest plot of skin-related adverse events rate in a meta-analysis of Achilles tendon ruptures.

NON, nonoperative treatment; MI, minimally invasive; M-H, Mantel-Haenszel; CI, confidence interval.


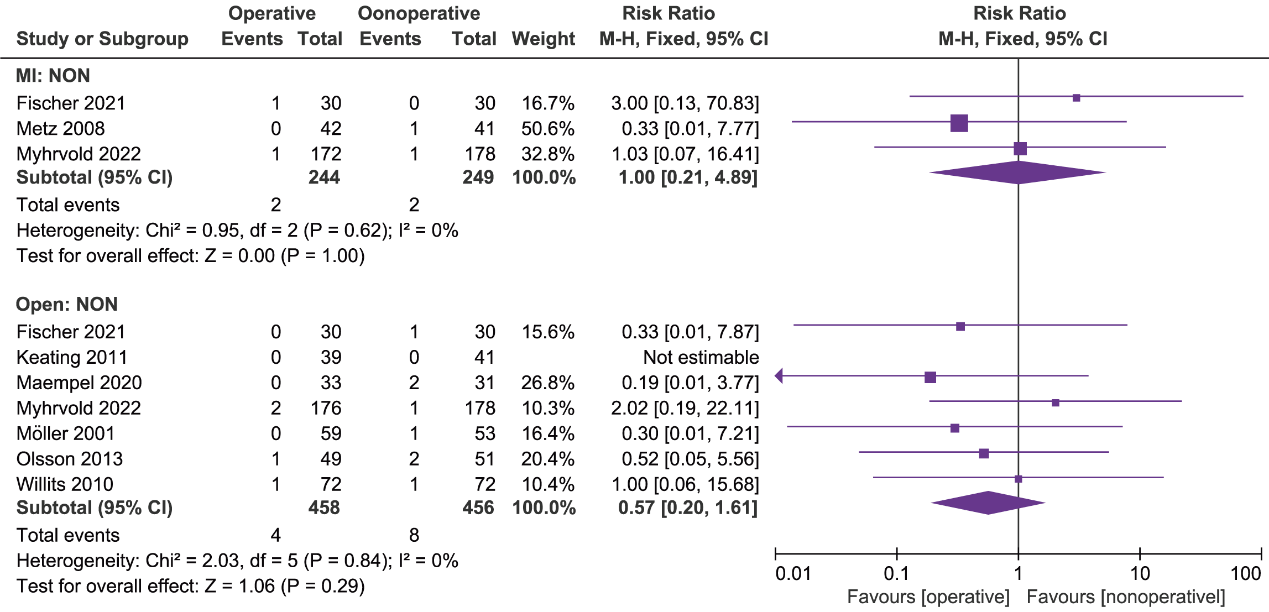


**Supplementary Figure 4** Forest plot of deep vein thrombosis rate in a meta-analysis of Achilles tendon ruptures.

NON, nonoperative treatment; MI, minimally invasive; M-H, Mantel-Haenszel; CI, confidence interval.


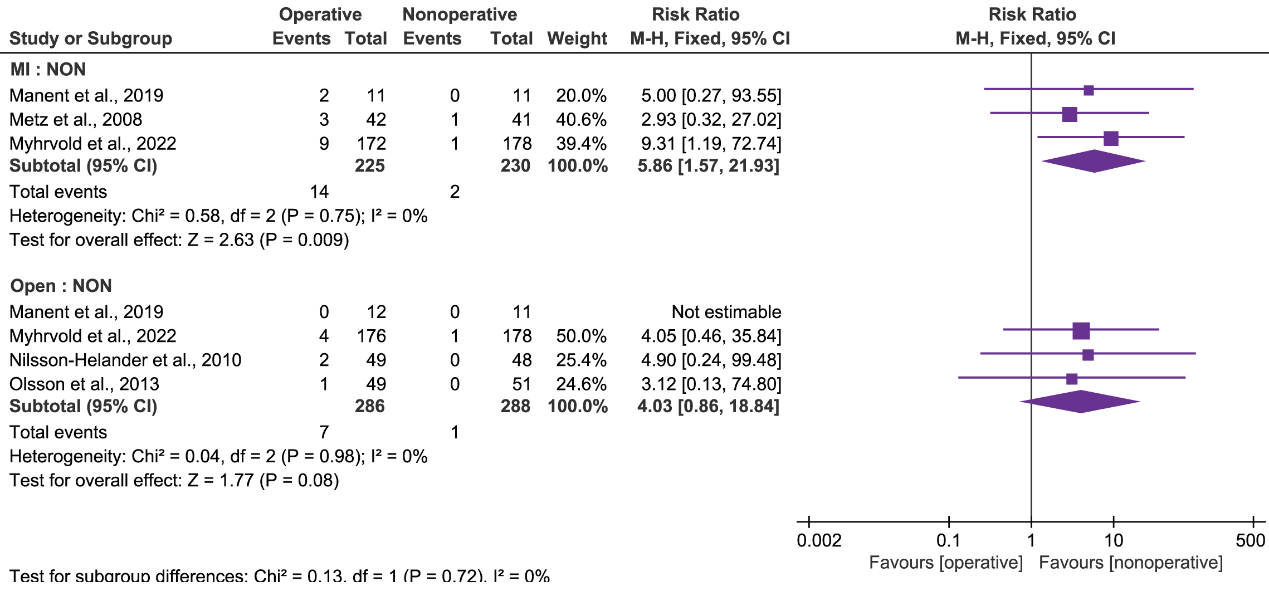


**Supplementary Figure 5** Forest plot of sural nerve lesions rate in a meta-analysis of Achilles tendon ruptures.

NON, nonoperative treatment; MI, minimally invasive; M-H, Mantel-Haenszel; CI, confidence interval.


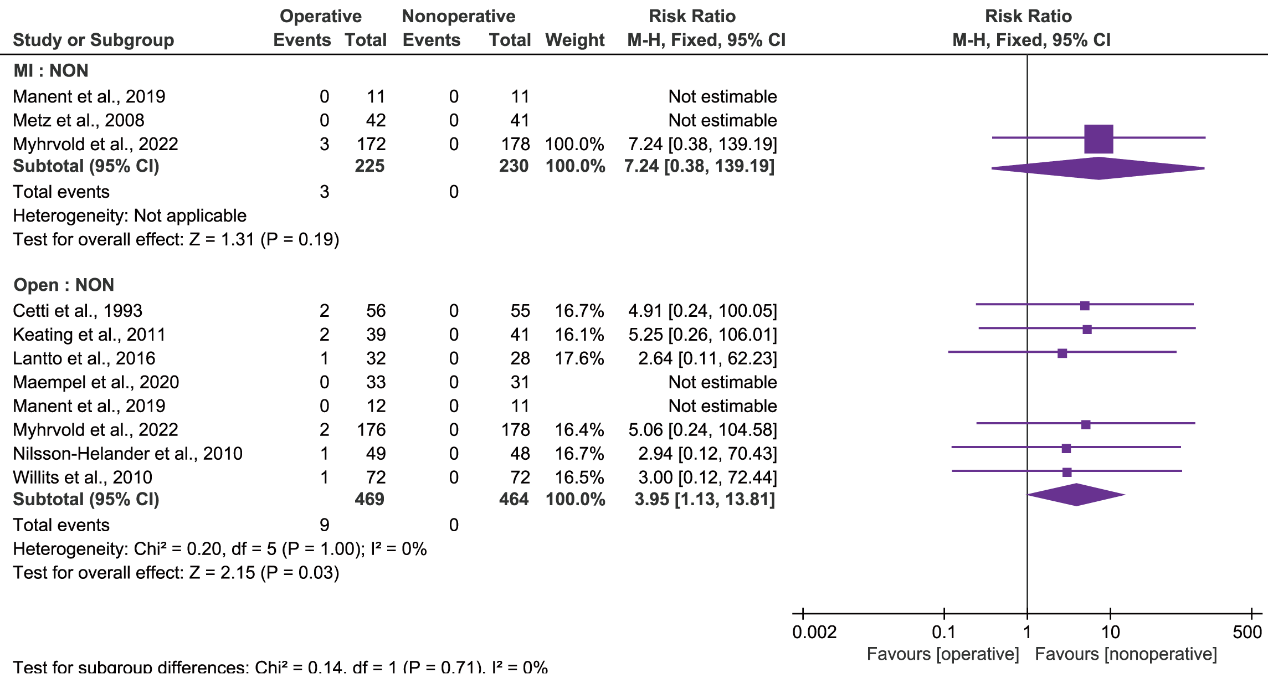


**Supplementary Figure 6** Forest plot of deep wound infection rate in a meta-analysis of Achilles tendon ruptures.

NON, nonoperative treatment; MI, minimally invasive; M-H, Mantel-Haenszel; CI, confidence interval.


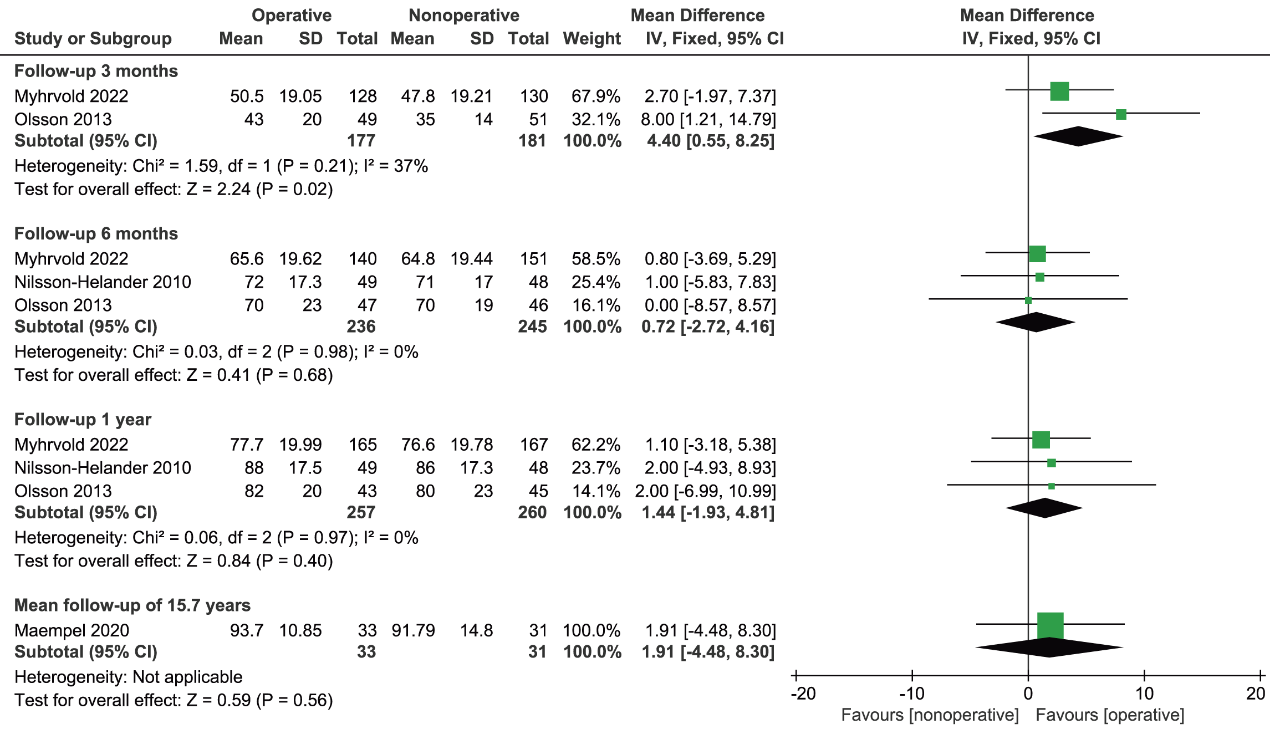


**Supplementary Figure 7** Forest plot of Achilles tendon rupture scores in a meta-analysis of Achilles tendon ruptures.

SD, standard deviation; CI, confidence interval.


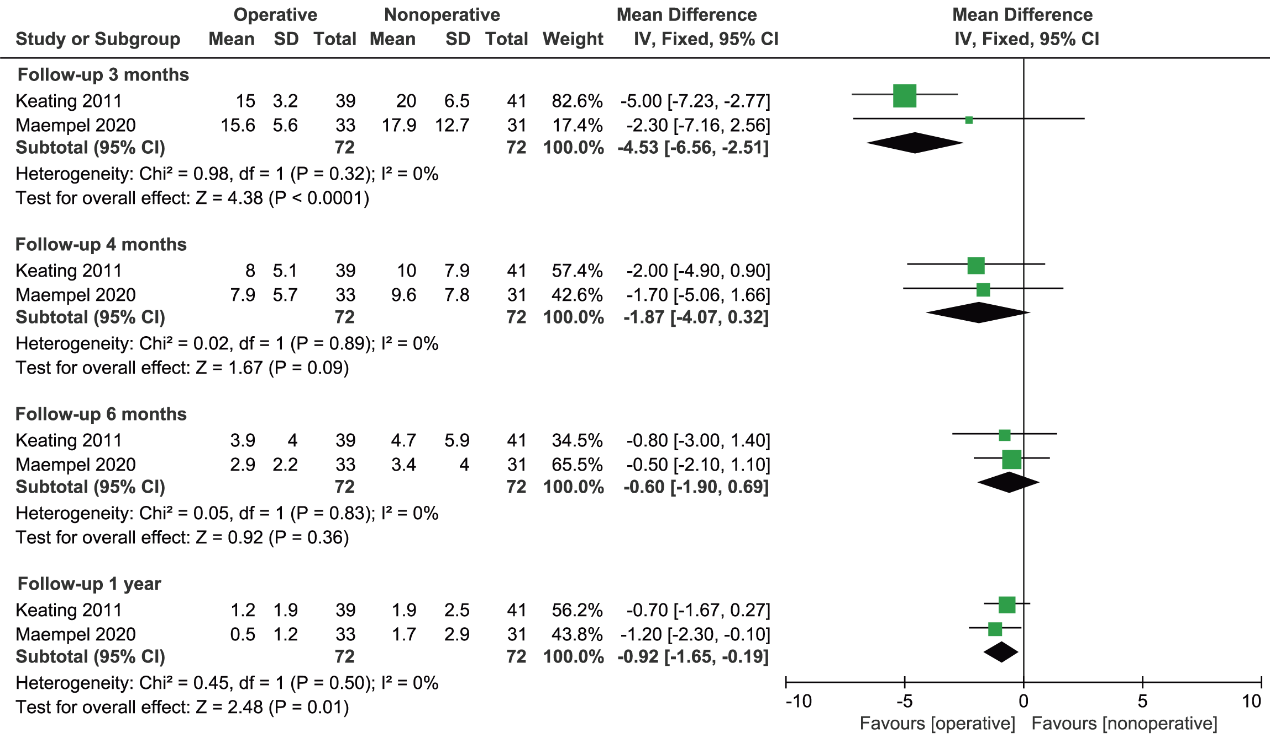


**Supplementary Figure 8** Forest plot of short musculoskeletal function assessment dysfunction score in a meta-analysis of Achilles tendon ruptures.

SD, standard deviation; CI, confidence interval.


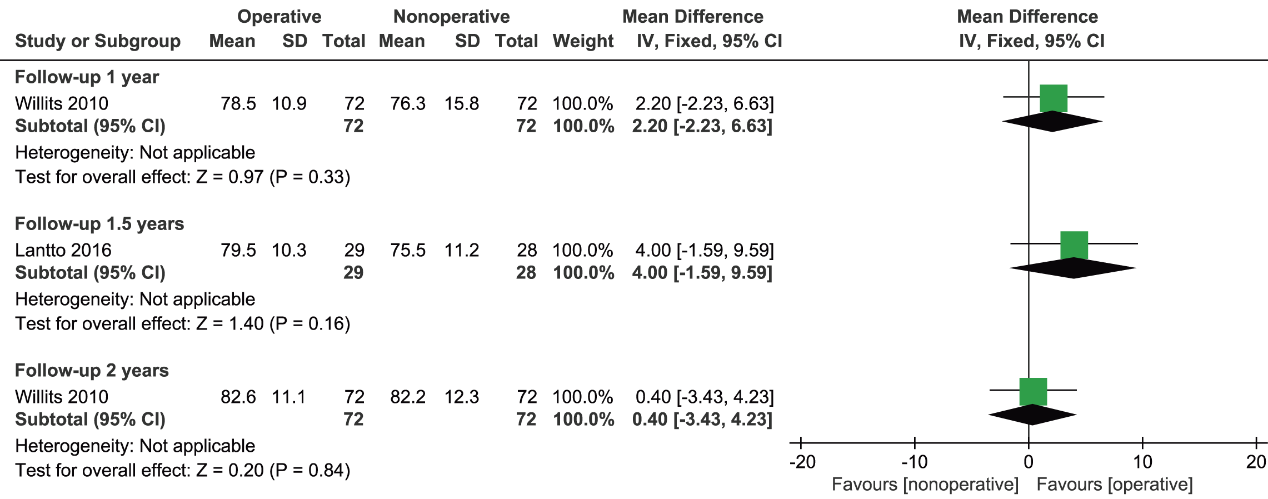


**Supplementary Figure 9** Forest plot of Leppilahti scores in a meta-analysis of Achilles tendon ruptures.

SD, standard deviation; CI, confidence interval.


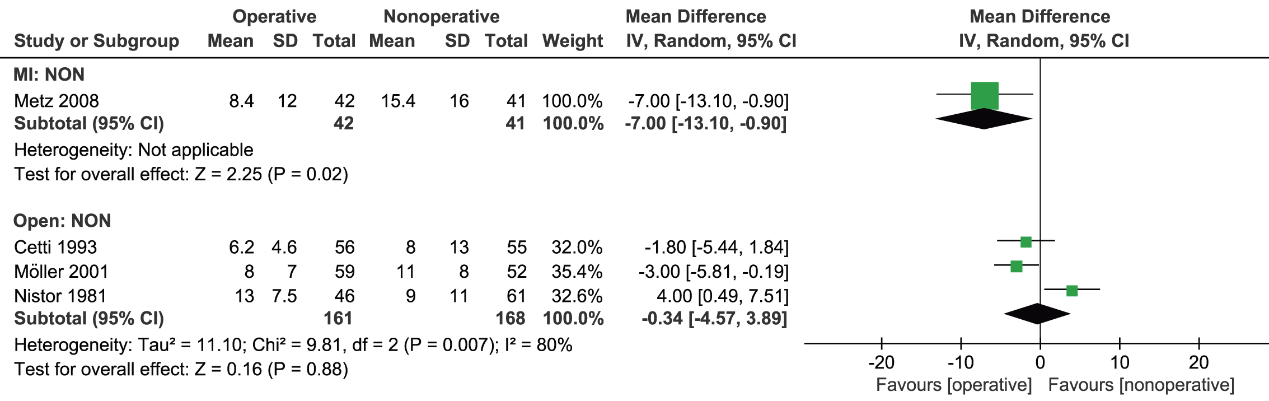


**Supplementary Figure 10** Forest plot of return to work in weeks in a meta-analysis of Achilles tendon ruptures.

NON, nonoperative treatment; MI, minimally invasive; SD, standard deviation; CI, confidence interval.
